# Standard and Variable Key Anatomical Structures for Safe Surgical Repair of Bochdalek Hernia: A Rare Case Series

**DOI:** 10.7759/cureus.6018

**Published:** 2019-10-28

**Authors:** Theodoros Mariolis-Sapsakos, Maria Zarokosta, Nikolaos Anastasiou, George Noussios, Dimitrios Filippou

**Affiliations:** 1 Anatomy, National and Kapodistrian University of Athens School of Medicine, Athens, GRC; 2 General Surgery, Agii Anargiri Oncological Hospital, Athens, GRC; 3 Thoracic Surgery, Agii Anargiri Oncological Hospital, Athens, GRC; 4 Physical Education and Sports Sciences, Aristotle University of Thessaloniki, Thessaloniki, GRC; 5 Surgery, Medical School of National and Kapodistrian University of Athens, Athens, GRC

**Keywords:** bochdalek hernia, diaphragm, hiatal hernia, congenital diaphragm defect, congenital hernia

## Abstract

Bochdalek hernia (BH) is a developmental defect in the posterolateral diaphragm, allowing herniation of abdominal contents into the thorax causing mechanical compression of the thoracic viscera. This type of hernia is rare in adults, usually asymptomatic and may be diagnosed incidentally in a routine chest X-ray.

The aim of the present retrospective study was to highlight the standard and important anatomical structures that are crucial to safe surgical repair of BH during laparotomy and thoracotomy by the placement of mesh graft, along with a short review of the existing evidence.

Records from 2005 to 2017 were reviewed to identify the patients with adult BH who underwent mesh repair through thoracotomy or laparotomy and evaluate the possible complications and results. Six patients were operated for adult BH with the above-mentioned techniques. Four underwent laparotomy and two were treated through thoracotomy. Mild fever was reported only in one patient. There was significant improvement in the symptoms of all patients and no recurrence was reported in the subsequent follow-up period.

Surgical treatment is strongly indicated for both symptomatic and asymptomatic patients. Surgeons’ in-depth knowledge of the anatomy of the diaphragm will ensure better outcomes for the patients.

## Introduction

Bochdalek hernia (BH) is a peculiar developmental defect in the posterolateral part of the diaphragm allowing herniation of the abdominal organs into the thoracic cavity [1-2]. The mechanical compression of the thoracic viscera caused by this pathology is associated with significant morbidity and mortality [3]. This type of herniation in adults is very rare, and its prevalence represents 0.17% to 6% among all diaphragmatic hernias [4]. Adults with BH usually present with symptoms of mechanical compression of abdominal or thoracic viscera, although they may remain asymptomatic on many occasions [5]. However, adults with this rare hernia may also present with life-threatening conditions, and thus surgery is indicated for both symptomatic and asymptomatic patients [2-3,6]. To date, only a few case series and case reports are available in the literature describing BH in adults. The present work, which is in line with the preferred reporting of case series in surgery (PROCESS) guidelines, aims to highlight the standard and variable key anatomical structures that may lead to safe surgical repair of BH during laparotomy and thoracotomy by the placement of mesh graft [7]. In addition, the present case series contains a meticulous review concerning the clinical manifestations and treatment options for BH in adult patients.

## Case presentation

Hospital records from 2005 to 2017 were reviewed to identify the patients with BH, who were treated by mesh repair of the diaphragm via thoracotomy or/and laparotomy. During this period, six patients were treated with the surgical intervention of left BH (Table 1). Four underwent laparotomy, while the other two were treated with thoracotomy. Both standard and variable anatomical structures that may contribute to the defect development in BH were identified in all patients, and both these surgical procedures were successfully performed. Therefore, the safe surgical technique that emphasizes on the anatomy of the diaphragm is adequately described.

**Table 1:** Patient data Patients diagnosed with adult BH and treated with thoracotomy or laparotomy and diaphragm repair by mesh placement BH, Bochdalek hernia; BMI, body mass index

Sex	Age	BMI (kg/m^2^)	Main symptom	Operation	Postoperative complications	Follow-up after 1 year
Male	28	23.2	Bowel strangulation	Laparotomy	None	-
Female	55	22	Indigestion	Laparotomy	None	-
Female	60	21.8	Abdominal discomfort	Laparotomy	None	-
Male	72	24.2	Incidentally detected during routine chest radiography	Thoracotomy	Fever	-
Male	61	21.5	Bowel strangulation	Laparotomy	None	-
Male	63	23	Incidentally detected during routine chest radiography	Thoracotomy	None	-

Preoperative care

**Figure 1:**
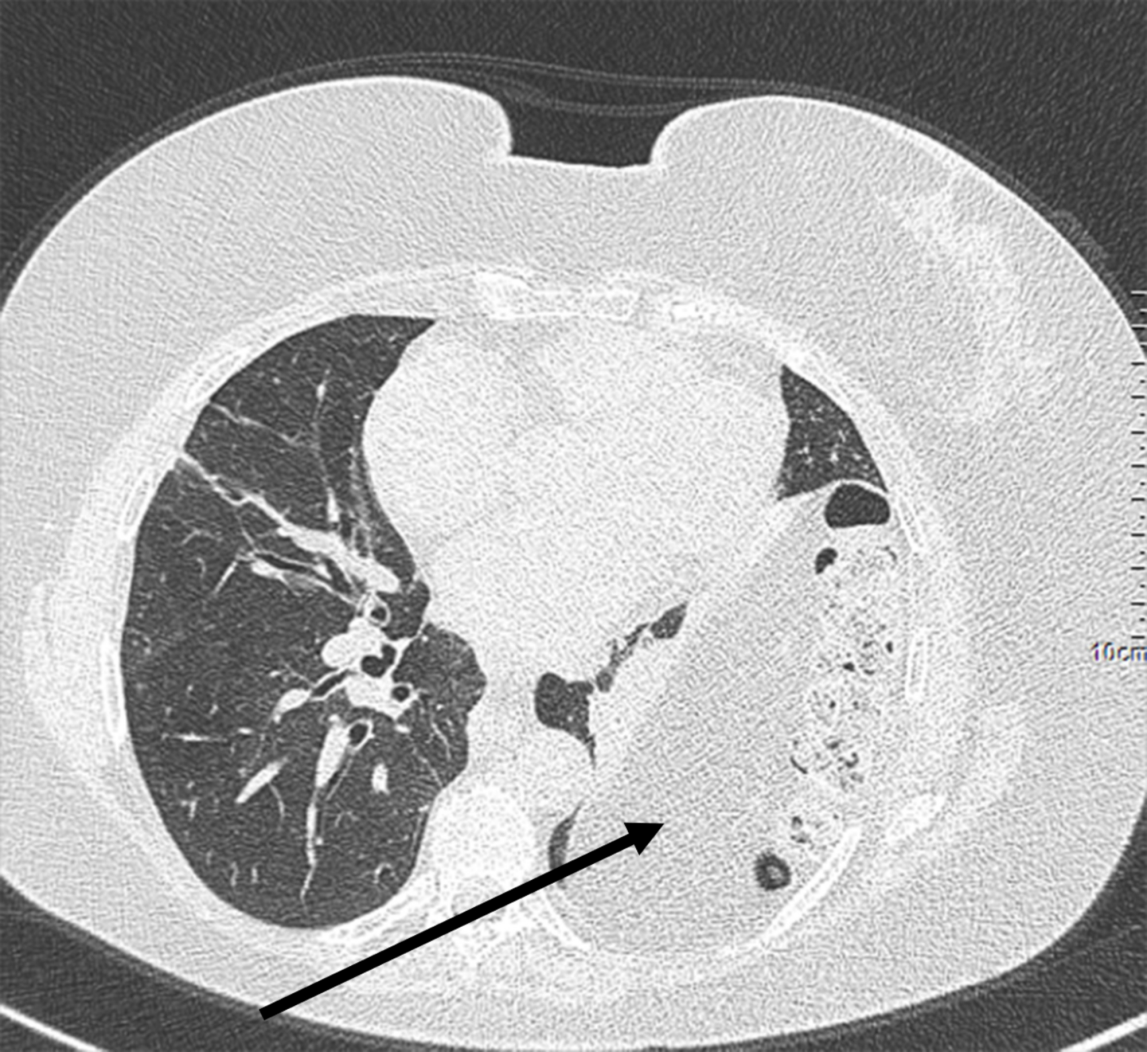
Thorax CT detected the splenic flexure and small intestine in the left chest, leading to mediastinal shift to the right CT, computed tomography

The majority of the patients in the present study presented with composite clinical symptoms, while two of them were clinically silent (Table 1). On physical examination, patients were detected with decreased breath sound on the left lower chest. Routine blood exams were normal. Chest X-rays and thorax computed tomography scans (CT) revealed that the splenic flexure and part of the small intestine had migrated into the left chest, leading to mediastinal shift to right (Figure 1). Patients with bowel strangulation underwent emergent laparotomy. Preoperatively, one dose of ceftriaxone had been administrated to all patients as chemoprophylaxis, two hours before the onset of the surgery followed by one more dose postoperatively.

Technique description

Thoracotomy was performed under general anesthesia with single lung ventilation. The patient was placed in the right decubitus position, due to the presence of left BH in both cases. During the operation, part of retroperitoneal adipose tissue was detected herniating into the left side of the thoracic cavity through the posterolateral defect of the diaphragm. Surgeons did not detect any hernia sac, which is common in adult BH cases. After removing the parietal pleura that enveloped the mass, the margins of the defect, which may constitute key anatomical structures through the operation, were revealed. The present procedure allows direct observation and exploration of the herniated contents. The central tendon of the diaphragm is the standard anterior margin of the defect, while the fascia of psoas major and quadratus lumborum muscle constitute the standard posterior margin. The lateral margins are formed by the lumbar and the costal portion of the diaphragm and their position may vary depending on the hernia’s size. After recognizing and preparing the related anatomic structures, the herniated tissue was returned to the abdominal cavity and a composite mesh graft was gently placed for closing adequately the large defect and reinforcing the diaphragm. Drainage was placed until the third postoperative day. 

All patients with gastrointestinal symptoms and abdominal discomfort underwent laparotomy. Surgeons performed a left subcostal incision to approach the posterolateral defect of the diaphragm. As in thoracotomy, surgeons recognized the important anatomical structures contributing to the hernia formation. The hernia content was returned to the abdominal cavity and then a flexible mess graft was gently fixed to the diaphragm with a continuous absorbable suture for the closure of the defect. Tubular drainage was placed in the left subdiaphragmatic area and was removed on the third postoperative day. 

A chest X-ray was routinely performed in all patients on the first postoperative day and was normal in all cases. Patients were discharged between the fifth and seventh postoperative days without complications with instructions and medications including analgesic and proton pump inhibitors (PPIs). Apart from the essential hygiene of the surgical wound, the patients were advised by the clinicians to use a stool softener for preventing probable postoperative constipation and subsequent intra-abdominal pressure.

Results

Hospital records revealed six patients treated for BH in whom a synthetic mesh was used to restore the diaphragm defect. Four out of six underwent laparotomy, while the rest two patients underwent thoracotomy. Surgeons used the central tendon of the diaphragm as the standard anterior margin of the Bochdalek defect, and the fascia of psoas major and quadratus lumborum as the standard posterior margin, to adequately remove all hernia contents and appropriately fix the composite mesh to the muscle. Diaphragmatic reconstruction with flexible mesh was performed in all patients. None of the patients presented any severe complications. One of the patients who underwent thoracotomy presented with fever on the first postoperative day, which was treated conservatively with levofloxacin and ceftriaxone administration. All of the patients were re-examined routinely two weeks postoperatively, and none presented any kind of complication. In follow-up after one year, all patients reported significant improvement of their symptoms while there was no evidence of recurrence.

## Discussion

BH is a rare congenital diaphragmatic defect first described by Bochdalek in 1848 [8]. This abnormality is caused by the failure of the posterolateral diaphragmatic foramen to fuse properly during the ninth or 10th week of gestation [2,9].

BH occurs in about one in 2200-12,500 live births and typically presents with acute cardiorespiratory failure during infancy [2,10]. Thus, the prevalence of BH in adults is 0.17% to 6% among all diaphragmatic hernias [4]. BH presents predominantly in males and almost invariably arises (80% to 90%) on the left side of the diaphragm [4,11-12]. 

BH in adults typically remains asymptomatic until compression symptoms occur later in life [2]. A major predisposing factor that provokes herniation, subsequent symptoms, and probable complications is the elevation of intra-abdominal pressure due to pregnancy, defecation, physical effort, and asthma attack [2,12]. Adults with BH and congenital diaphragmatic hernia often demonstrate pulmonary symptomatology with chronic lung disease, but also neurocognitive delay, gastroesophageal reflux disease (GERD), chest wall deformity, poor growth, and hernia recurrence. However, adult patients more frequently suffer from non-specific digestive and respiratory symptoms including chronic dyspnea, recurrent chest pain, pleural effusion, abdominal pain, vomiting, and postprandial fullness [2,11]. 

Nevertheless, adult BH is closely associated with a mortality rate of 5% due to the possibility of herniation of abdominal organs and other complications [2,6,13]. Most commonly, patients present with emergent conditions such as syncope, bowel herniation acute pancreatitis, and sepsis [2,3,14-16]. BH in adults is usually misdiagnosed clinically and prompt diagnosis is mainly based on radiological findings [6,11]. Asymptomatic cases are usually incidentally detected during routine chest radiography [4]. Chest X-rays (lateral and frontal view) are a useful diagnostic tool in addition to thorax CT especially in doubtful cases [5].

Regardless of symptoms, timely and accurate diagnosis and surgical treatment are crucial [2]. The first successful treatment was performed in 1901 by Aue, but the optimal surgical strategy remains controversial [11,17]. In fact, the surgical approach depends on the patient’s clinical condition and the surgeon’s experience [4,17]. Although laparotomy is the most frequently used approach (38%), thoracotomy is therapeutically equal, and in some cases even essential for reducing the abdominal contents and repairing the posterolateral diaphragmatic defect [4,11]. Recently, minimally invasive techniques, such as laparoscopy and thoracoscopy have been developed [1,5-6,11,13]. Lamentably though, the pneumoperitoneum technique during these operations may lead to progression of herniation and subsequent cardiorespiratory failure, in addition to probable intraoperative or even postoperative pneumothorax [2]. Hence, in our institution, laparotomy and thoracotomy are preferred as well as the use of composite, flexible mesh graft for the surgical repair of the adult BH and reinforcement of the diaphragm. Mesh graft placement is well tolerated and strongly recommended when the size of the foramen defect exceeds 20-30 cm^2^ [2]. According to our opinion as surgeons and anatomists, surgeons should always try to identify the important anatomical structures that contribute to the Bochdalek defect in detail. Due to their morphological structure, the central tendon of the diaphragm anteriorly and the fascia of psoas major and quadratus lumborum posteriorly maintain their anatomical position independently from the BH size. Therefore, these standard anatomical structures guide surgeons intraoperatively and help them restore the herniated viscera, while they also constitute safe positions for the consequent placement of the mesh graft. The lateral margins of the Bochdalek defect are formed by the lumbar and the costal portion of the diaphragm and their position may vary depending on the hernia’s size since loosening due to increasing pressure by the hernia content. Surgeons are taught to detect these variable margins as well, in order to secure surgical repair’s efficiency and safety. Nevertheless, the limitation of the study is the restricted sample of patients due to the scarcity of adult BH.

## Conclusions

BH in adults is a rare congenital pathology with significant morbidity and mortality. Surgical treatment is strongly indicated for both symptomatic and asymptomatic patients. Surgeons’ in-depth knowledge of the anatomy of the diaphragm will ensure better outcomes for the patient.
